# Broad-Spectrum Amino Acid Transporters ClAAP3 and ClAAP6 Expressed in Watermelon Fruits

**DOI:** 10.3390/ijms20235855

**Published:** 2019-11-22

**Authors:** Tianran Shi, Vijay Joshi, Madhumita Joshi, Stanislav Vitha, Holly Gibbs, Kehua Wang, Sakiko Okumoto

**Affiliations:** 1 Department of Soil and Crop, Texas A&M, College Station, TX 77843, USA; shitianran123@163.com; 2 College of Grassland Science and Technology, China Agricultural University, Beijing 100083, China; kehwang@cau.edu.cn; 3 Texas A&M AgriLife Research and Extension Center, Uvalde, TX 78801, USA; Vijay.Joshi@ag.tamu.edu (V.J.); Madhumita.Joshi@ag.tamu.edu (M.J.); 4 Microscopy and Imaging Center, Texas A&M, College Station, TX, 77843, USA; stanvitha@tamu.edu (S.V.); hgibbs@exchange.tamu.edu (H.G.)

**Keywords:** nitrogen, amino acids, citrulline, watermelon, transport

## Abstract

Watermelon fruit contains a high percentage of amino acid citrulline (Cit) and arginine (Arg). Cit and Arg accumulation in watermelon fruit are most likely mediated by both *de novo* synthesis from other amino acids within fruits and direct import from source tissues (leaves) through the phloem. The amino acid transporters involved in the import of Cit, Arg, and their precursors into developing fruits of watermelon have not been reported. In this study, we have compiled the list of putative amino acid transporters in watermelon and characterized transporters that are expressed in the early stage of fruit development. Using the yeast complementation study, we characterized ClAAP3 (Cla023187) and ClAAP6 (Cla023090) as functional amino acid transporters belonging to the family of amino acid permease (AAP) genes. The yeast growth and uptake assays of radiolabeled amino acid suggested that ClAAP3 and ClAAP6 can transport a broad spectrum of amino acids. Expression of translational fusion proteins with a GFP reporter in *Nicotiana benthamiana* leaves confirmed the ER- and plasma membrane-specific localization, suggesting the role of ClAAP proteins in the cellular import of amino acids. Based on the gene expression profiles and functional characterization, ClAAP3 and ClAAP6 are expected to play a major role in regulation of amino acid import into developing watermelon fruits.

## 1. Introduction

Watermelon (*Citrullus lanatus*) by far accumulates the highest amount of arginine (Arg) and citrulline (Cit) among commonly consumed fruits and vegetables, at >40% of total amino acids in its fruits. There is an interest in increasing Cit and Arg content in watermelon fruits for nutraceutical purposes. Oral uptake of Cit has been suggested to increase the bioavailability of plasma Arg, which, in turn, is essential for NO production [1,2]. NO is synthesized by the family of nitric oxide synthases from Arg, yielding Cit as a co-product. NO is a signaling molecule and plays an essential role in numerous physiological processes, including blood vessel vasodilatation [1,2,3]. The importance of citrulline in human health has also been reviewed extensively [1,4,5]. Cit is also a potent scavenger of hydroxyl radicals, having higher scavenging activities compared to mannitol, proline, and glycine betaine [6,7]. Therefore, understanding the mechanisms of Cit accumulation in watermelon could lead to improved stress tolerance, as well as an increased nutraceutical value.

High-throughput sequencing of the watermelon genome and transcriptome has identified several genes involved in Arg and Cit biosynthesis in fruits, as well as their regulation during the development of fruits. For example, whole-genome sequence and RNAseq from developing fruits of watermelon revealed that the families of genes encoding the enzymes involved in Arg and Cit metabolism were expanded in watermelon compared to *Arabidopsis* [8]. Moreover, the transcriptome data showed that Cit catabolism is downregulated during fruit development, suggesting that Cit accumulation is at least partially accomplished via altered metabolism in developing fruits [9,10].

Generally, developing sink organs such as seeds and fruits are dependent on nitrogen (N) supply via the phloem to support their vigorous growth. *Cucurbitaceous* plants are known to contain Arg and Cit, as well as their precursors such as ornithine (Orn), glutamine (Gln), and glutamate (Glu) at a high concentration in leaves and the phloem, suggesting that these amino acids are the primary N carriers in *Cucurbitaceous* species [11,12]. Because it is well established that the quantity and composition of amino acids in the phloem, as well as the strength of amino acid import activity at the sink organ, are correlated with the N content in the sink organ [13,14,15], understanding of the amino acid transporters involved in fruit loading could open new opportunities to engineer a pathway for fruit Cit content improvement. However, amino acid transporters in watermelon fruits have not been yet characterized.

Here, we have curated the amino acid transporters from watermelon by querying the recent genomic data [8]. Further, using publicly available datasets, we have identified three members of the amino acid permease family expressed in fruits, two of which we have confirmed as amino acid importers. Our data suggest both transporters recognize a broad range of amino acids, except for some differences in the substrate specificities.

## 2. Results

### 2.1. Identification of Amino Acid Importers from Watermelon

In order to identify the amino acid transporter genes from watermelon, the predicted protein sequences from cultivar 97103 [8] was queried using the consensus sequences built from known amino acid transporter families (lysine histidine transporter (LHT), proline transporter (ProT), GABA transporter (GAT), amino acid permeases (AAP), aromatic and neutral amino acid transporter (ANT), auxin transporter (AUX), putrescine transporter (PUT), and cationic amino acid transporter (CAT)). The phylogenetic analysis of *Arabidopsis* and watermelon amino acid transporters revealed at least one member in each family (Table 1, Figure S1).

**Figure 1:**
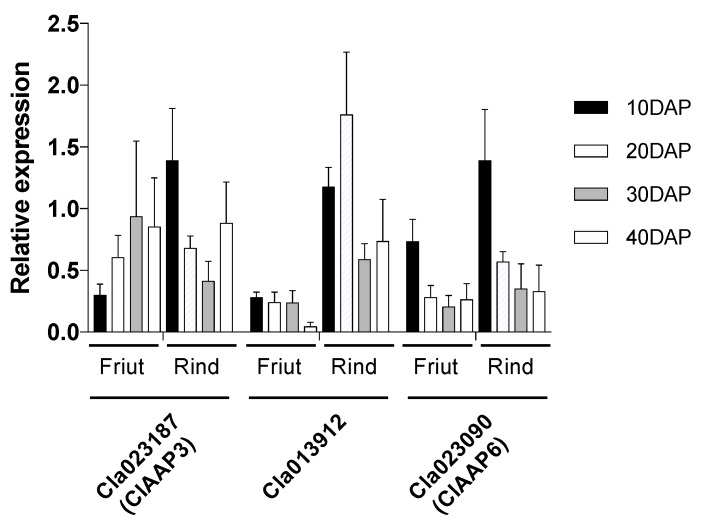
Expression of putative amino acid transporters in the fruit tissues. The relative expression level of each gene was measured by qRT-PCR using gene-specific primers shown in Table S1. RNA samples were extracted from fruits and rind tissues at 10, 20, 30, and 40 days after pollination (DAP). The expression levels were normalized using actin gene. The data were obtained from three biological replicates.

Next, we have attempted to identify the amino acid transporters expressed in the developing fruit tissues by intersecting the list of transporters with the publicly available transcriptome data of fruit and rind tissues [9,16]. Among those found to be expressed in the fruit tissue, we have focused on the members of the AAP family since previous studies revealed that the AAPs from other plant species transport a broad spectrum of amino acids, including Cit [17,18,19,20]. Three genes, Cla023090, Cla023187, and Cla013912, have been identified based on their expression in fruit and rind tissues. Quantitative RT-PCR results further confirmed the results from RNAseq studies (Figure 1). All genes were expressed in both fruit and rind tissues throughout the developmental stages tested (10 to 40 days after pollination). Cla023187 expression in fruits increased at the later stages (20, 30 and 40 days after pollination). The expression of Cla023090 and Cla013912 was also developmentally regulated, with stronger expression found in earlier developmental stages of fruits in both flesh and rind tissues.

### 2.2. ClAAP3 and ClAAP6 Complements the Growth of Yeast Cells Deficient in Amino Acid Uptake

**Figure 2:**
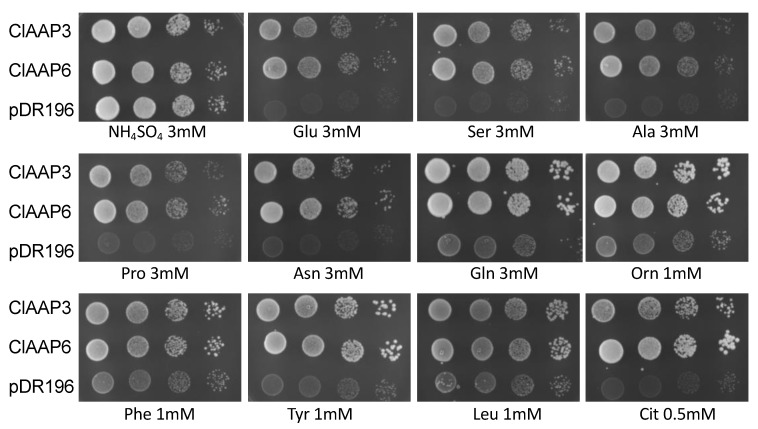
ClAAP3 (Cla023187) and ClAAP6 (Cla023090) complements the growth of 22Δ10α on amino acids. 22Δ10α cells carrying either *ClAAP3*, *ClAAP6,* or the empty vector (pDR196) were grown for 3–4 days on a minimal medium containing either NH_4_SO_4_ or amino acids as the sole nitrogen source at the concentrations indicated. Cells were diluted tenfold from left to right, with the left column representing OD_600_ = 1.0.

Yeast growth complementation assays were performed to investigate whether the newly identified ClAAP genes (Cla023090, Cla023187, and Cla013912) are indeed encoding amino acid transporters. We used a *Saccharomyces cerevisiae* strain 22Δ10α that had lesions in all endogenous amino acid transporters and grows poorly on all proteinogenic amino acids except for Arg [21]. Vectors carrying putative ClAAP expression constructs were used to transform mutant strain 22Δ10α. Cells expressing Cla023187 and Cla023090 coding sequences were able to grow on a medium containing Ala, Asn, Gln, Glu, Pro, Ser, Tyr, Phe, and Leu, as well as non-proteinogenic amino acids Cit and Orn as the sole source of nitrogen (Figure 2), whereas Cla013912 failed to complement the growth of 22Δ10α on any of the amino acids tested (data not shown). From these results, we have concluded that Cla023187 and Cla023090 function as amino acid transporters. Based on their primary sequence similarity to the respective *Arabidopsis* AAPs (Figure S1), we named Cla023187 and Cla023090 as ClAAP3 and ClAAP6, respectively.

### 2.3. ClAAP3 and ClAAP6 are Broad-Spectrum Amino Acid Transporters

**Figure 3:**
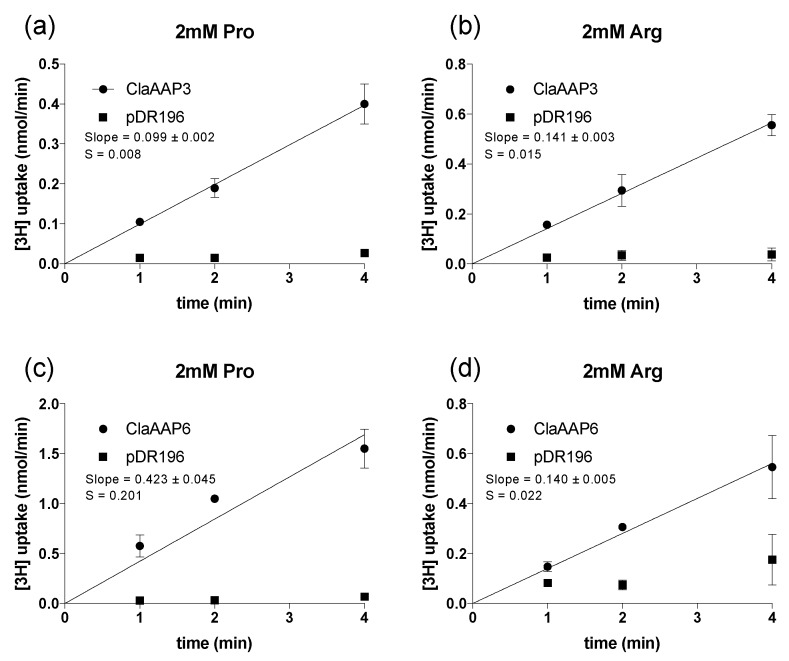
Uptake of [^3^H] labeled amino acids by yeast 22∆10α cells. (**a**,**b**) Time-dependent uptake of [^3^H]Pro (**a**) or [^3^H]Arg (**b**) into the cells expressing ClaAAP3 (circles) or empty vector (squares). (**c**,**d**) Time-dependent uptake of [^3^H]Pro (**c**) or [^3^H]Arg (**d**) into the cells expressing ClaAAP6 (circles) or empty vector (squares). The uptake of [^3^H]-labeled amino acids was measured at 2 mM concentration. Each data point represents three biological replicates. Error bars represent standard deviation (*n* = 3). The standard error of the regression (S) is shown for each linear fit.

**Figure 4:**
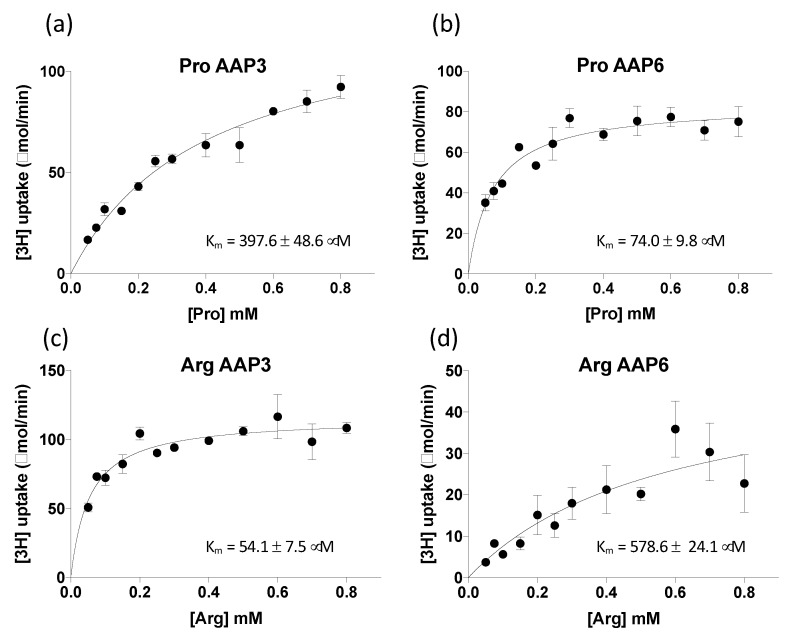
Concentration dependence of Pro and Arg uptake for ClAAP3 and ClAAP6. (**a**) and (**b**) [^3^H] Pro uptake into yeast 22∆10α cells expressing ClAAP3 (**a**) or ClAAP6 (**b**). (**c**) and (**d**) [^3^H] Arg uptake into yeast 22∆10α cells expressing ClAAP3 (**c**) or ClAAP6 (**d**). Values obtained from cells expressing the empty vector were used to normalize each data point. Michaelis–Menten kinetics were used to calculate *Km* values. Error bars represent standard deviation (*n* = 3).

To understand the kinetics and substrate specificity of ClAAP3 and ClAAP6, we monitored the uptake of ^3^[H]- labeled amino acids into the 22Δ10α cells expressing ClAAP3 or ClAAP6. Both ClAAP3 and ClAAP6 were able to transport ^3^[H]- labeled Pro and Arg (Figure 3), two amino acids that accumulate during early and late stages of watermelon fruit growth, respectively [22]. Amino acid uptake stayed linear for the first four minutes of the experiment (Figure 3), similar to previous reports [17,18,21]. Arg transport into the cells carrying the empty vector (22Δ10α–pDR196) was minimal compare to those expressing ClAAP3 and ClAAP6 (Figure 3 and Figure 4), even though 22Δ10α-pDR196 can grow on a medium containing Arg as the sole nitrogen source [21]. It is possible that the growth of 22Δ10α–pDR196 is supported by a mechanism that bypasses amino acid transport, such as activity of extracellular deaminase that is produced in response to Arg in other fungal species [23,24]. Since we did not observe significant uptake activity for ^3^[H]-Arg in 22Δ10α–pDR196 under our condition, we proceeded to determine biochemical properties of both Pro and Arg transport using 22Δ10α cells.

*Km* values of ClAAP3 and ClAAP6 for Pro were 397.6 ± 48.6 μM and 74.02 ± 9.8 μM, respectively, suggesting a greater affinity of ClAAP6 for Pro than ClAAP3 (Figure 4). On the other hand, ClAAP3 had a higher affinity for Arg (*Km* = 54.1 ± 7.5 μM) than ClAAP6 (*Km* = 578.6 ± 24.1 μM). Similar to AAPs from other species [19,25], the uptake activity of ClAAP3 and ClAAP6 was sensitive to protonophore carbonyl cyanide *m*-chlorophenyl hydrazone (CCCP), indicating that ClAAAP3 and ClAAP6 are H^+^-symporters (Figure S2).

**Figure 5:**
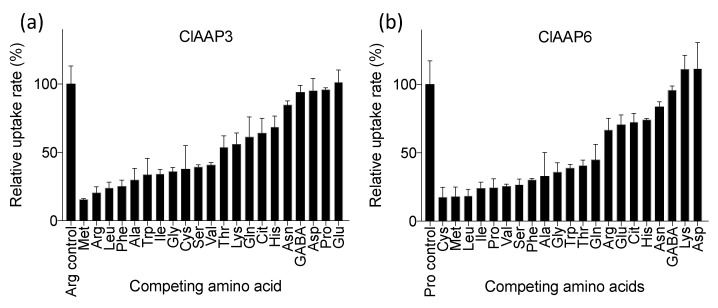
Effects of competing amino acids to ClAAP3 and ClAAP6 mediated amino acid uptake. (**a**) Effects of competing amino acids on [^3^H]-Arg uptake by ClAAP3. (**b**) Effects of competing amino acids on [^3^H]-Pro uptake by ClAAP6. In both experiments, [^3^H]- labeled amino acids were supplied at 1 mM concentration, whereas the competing unlabeled amino acids were supplied at five-fold concentrations (i.e., 5 mM). The values are expressed as % of cells that did not receive any competing amino acids. Error bars represent standard deviation (*n* = 3).

Both the yeast growth and the radiolabeled amino acid uptake assays suggest that ClAAP3 and ClAAP6 are capable of transporting a broad spectrum of amino acids. Competition assays were performed to evaluate the affinities of ClAAP3 and ClAAP6 for different amino acids. Yeast cells expressing ClAAP3 or ClAAP6 were co-incubated with ^3^[H]-labeled amino acids for which each transporter has a high affinity (Arg for ClAAP3 and Pro for ClAAP6, Figure 4) in the presence or absence of competing amino acids. The results suggest that both transporters recognize non-polar (aliphatic and aromatic) amino acids. They differ, however in the relative affinity to some key amino acids; Arg is one of the preferred substrates for ClAAP3, whereas it is not a preferred substrate for ClAAP6 (Figure 5). Pro and Glu, both of which can serve as the precursor for Orn and Cit, compete poorly for ClAAP3, but were well recognized by ClAAP6. Cit, a non-protein amino acid that accumulates at a high concentration during watermelon fruit maturation, is recognized by both ClAAP3 and ClAAP6.

### 2.4. Subcellular Localization of ClaAAP3 and ClaAAP6

**Figure 6:**
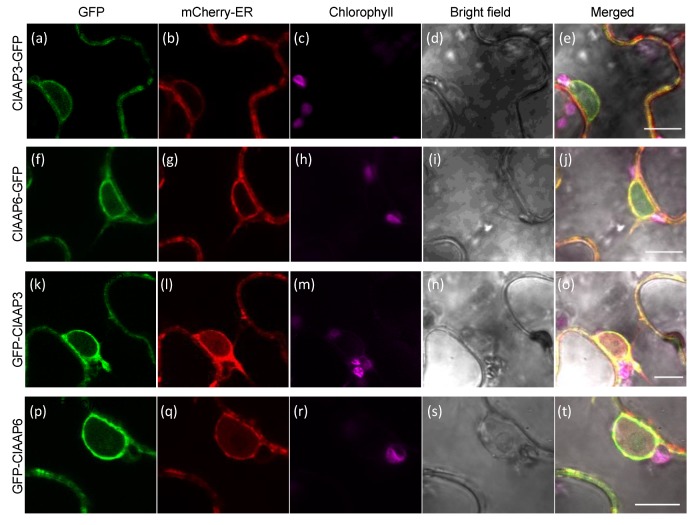
Subcellular localization of GFP-tagged ClAAP3 and ClAAP6 The signal from GFP (**a**,**f**,**k**,**p**); mCherry (**b**,**g**,**l**,**q**); Chlorophyll (**c**,**h**,**m**,**r**); bright field (**d**,**i**,**n**,**s**) channels, and the merged picture (**e**,**j**,**o**,**t**) are shown. (**a**–**e**): *Nicotiana benthamiana* leaf epidermal cells co-expressing ClAAP3-GFP and mCherry-HDEL (ER marker). (**f**–**j**) *Nicotiana benthamiana* leaf epidermal cells co-expressing ClAAP6-GFP and mCherry-HDEL. (**k**–**q**) *Nicotiana benthamiana* leaf epidermal cells co-expressing GFP-ClAAP3 and mCherry-HDEL. (**p**–**t**) *Nicotiana benthamiana* leaf epidermal cells co-expressing GFP-ClAAP6 and mCherry-HDEL Scale bars: 10 μm.

Subcellular localization of ClAAP3 and ClAAP6 were performed by using translational fusions with GFP in *Nicotiana benthamiana* leaves. Each AAP gene was translationally fused with GFP on either N’- and C’- side, since previous studies have shown that depending on the location of targeting motifs, both C or N terminal fusions can mislocalize [26,27]. The results showed that both fusions localize to the structure resembling perinuclear ER membrane and the periphery of the cells, presumably the plasma membrane (Figure 6a,f,k,p). The signal from the plasma membrane was slightly stronger with N’-fusions (Figure 6k,p). Co-expression of GFP fusion constructs and an ER lumen marker (mCherry-HDEL, [28]) showed a considerable overlap of GFP signal with the signal from the mCherry marker, suggesting that the GFP fusions were localized to the ER membrane (Figure 6e,j,o,t). Similar results were obtained when ClAAP3- and ClAAP6-GFP fusions were expressed in protoplasts isolated from young watermelon fruits (Figure S3).

## 3. Discussion

We have identified two amino acid transporters, ClAAP3 and ClAAP6, that are expressed in developing fruits of watermelon. Substrate specificities of the two transporters demonstrated non-overlapping patterns, with ClAAP3 being an efficient transporter for basic amino acids. These results are consistent with the phylogenetic analysis, which showed that ClAAP3 belongs to the same subclade as AtAAP3 and AtAAP5, which have higher affinities to basic amino acids (Figure S1) [17,18].

Watermelon fruits accumulate high amounts of Cit and Arg in both rind and fruits [7]. The accumulation is developmentally controlled; Cit concentration dramatically increases as the fruit ripens (2–5 folds compared to immature fruits), while Arg, the precursor of Cit, also accumulates [8,22,29]. The mechanisms for such a high level of Cit accumulation is not entirely understood. Transcriptomic studies in developing fruits have shown that the enzymes involved in Cit catabolism are down-regulated during fruit maturation, suggesting that the slower Cit catabolism is partially responsible for the high level of Cit in mature fruits [8,9]. On the other hand, it has also been suggested that Cit derived from source leaves play a role in Cit accumulation in fruits, as Cit functions as a significant part of organic N in Cucurbitaceous species [11,12]. Recently it has been shown that Cit accumulation is positively correlated with Arg during watermelon fruit ripening [10]. Within this context, it is notable that the expression of ClAAP3, which transports both Arg and Cit, increases as the fruit ripens (Figure 1). Whether ClAAP3 plays a specific role in Arg and Cit homeostasis remains to be established.

The substrate specificity of ClAAP6 is not consistent with the potential role in Arg and Cit accumulation in the fruits. The expression of ClAAP6 in developing fruits is higher at an earlier stage of development, which also makes this transporter an unlikely candidate for Arg and Cit accumulation at the later stage of development. On the other hand, amino acids such as Gln and Pro accumulate at a higher level in immature watermelon fruits [22], both of which are well transported by ClAAP6 (Figure 5b). Therefore, ClAAP6 might be involved in the uptake of these amino acids at the earlier stages of fruit development. In both cases, further studies are needed to evaluate whether the increased expression of ClAAP genes increases the level of ClAAP proteins and transport activity.

GFP-tagged ClAAP3 and ClAAP6 localized mainly to the ER membrane when expressed transiently in the leaves of *Nicotiana benthamiana* and protoplasts isolated from young watermelon fruits. Localization of N’-terminal fusion is similar to the C’- fusion, with a stronger signal from the plasma membrane. Similar results have been previously observed with rice AAP proteins, which reported attenuation of GFP signal fused to the C’ terminus, likely due to the topology of AAP that exposes C’-termini to the acidic apoplast [20]. Other members of the AAP family have also been reported to localize to the ER membrane when expressed transiently [30,31]. However, these observed localization patterns could be artefactual due to the expression systems used. ClAAP proteins might require an accessory protein to exit the ER, as demonstrated for other membrane proteins [32,33,34,35]. Our recent work showed that, indeed, the localization of an amino acid transporter was not correctly replicated in the cell type that does not typically express this transporter [36]. Currently, we do not know the cell type that expresses ClAAP3 and ClAAP6 genes within the developing fruits. Generating transgenic lines expressing transcriptional fusion constructs using native promoters will help in determining their tissue specificities. For C’- fusion, pH-stable fluorescent proteins would be needed to estimate the signals at the plasma membrane accurately [20].

We were unable to show any amino acid transport activity for Cla013912, although the primary sequence of this transporter is very similar to that of ClAAP3 (82.0% identity). This could be due to the localization of the protein to the membrane other than the plasma membrane, as was observed for different amino acid transporters from plants [25,37]. The possibility of Cla013912 functions as amino acid transporters in other intracellular membranes remains to be validated.

## 4. Materials and Methods

### 4.1. Identification of Amino Acid Transporters from the Watermelon Genome

Peptide databases from plants representing diverse groups of embryophytes were searched with known amino acid transporters (AtAAP1, AtLHT1, ANT1, AtProT1, AtUMAMIT18, CAT1, and BAC1), using BLAST-P search (version 2.2.29, [38]). The obtained sequences were aligned with MUSCLE [39], and the consensus sequences were built using the HMMER algorithm [40]. The watermelon protein sequence database [8] was queried with a consensus sequence using the HMMERsearch to obtain potential AATs from watermelon. As expected, the protein list of members of the AAAP superfamily (AAP, LHT, ANT, ProT/GAT family) was cross-detected by BLAST-P due to the sequence homology within the superfamily. The clades within the AAAP superfamily were resolved using OrthoMCL to identify the members of each family [41]. In total, we identified 54 putative amino acid importers from watermelon (Table 1). The amino acid transporters from *Arabidopsis* and watermelon were aligned using Muscle [39], and the phylogenetic tree was built using the maximum likelihood method [42].

### 4.2. DNA Constructs

The coding sequences (e.g., the starting codon to the stop codon) of Cla023090, Cla023187 and Cla013912 were synthesized with the Gateway adapter sequences, attB1 (5′- ggggacaagtttgtacaaaaaagcaggcttc -3′) and attB2 (5′- gacccagctttcttgtacaaagtggtcccc -3′) on 5′- and 3′- ends, respectively. The fragments were then cloned into pDONRZeo-f1 vector [43]. For yeast assays, the genes were transferred to the destination vectors pDR196-Ws [44]. For the expression ClAAP-GFP (e.g., C’-GFP fusions) in plants, the coding sequence without the stop codon was amplified using Table S1. Once the construct was sequenced and confirmed PCR error-free, each coding sequence was fused with the 35S promoter and omega leader sequence on the 5′- side and GFP, Riblose-1,5-Bisphosphate Carboxylase oxygenase Small chain (RBCS) terminator and kanamycin-resistant cassette on the 3′- side through a one-pot reaction as described in [45], creating pGGZ-ClAAP3-GFPand pGGZ-ClAAP6-GFP. The components used for the reactions were; 35S promoter, pGGA004; omega leader sequence, pGGB002; GFP, pGGD001; RBCS terminator, pGGE001; kanamycin-resistant cassette, pGGF007; destination vector, pGGZ001; as described in [45]. For N’- YFP fusions, the ClAAP genes in pDONRZeo-f1 vector was cloned into to PGWB506 [46].

### 4.3. Plant Material, Growth, Transformation, and Analysis

*Nicotiana benthamiana* plants were grown in soil culture in a growth chamber kept at 25 °C day/20 °C night, under a 16/8 h day/night cycle. Plants of watermelon (*Citrullus lanatus* (Thunb.) Matsum. & Nakai) cv. Charleston Gray were grown in the field in three replications at Texas A&M AgriLife Research Center, Uvalde, TX using standard horticultural practices. Tissue samples were collected in triplicate from fruits (flesh) and rind tissues at 10, 20, 30, and 40 days after pollination in the field. For transient expression assay in protoplasts, watermelon plants were grown in the field in College Station, TX, using standard practices.

### 4.4. Yeast Assays

The yeast strain 22Δ10α [21] was transformed with pDR196-Ws vector [44] carrying ClAAP3, ClAAAP6, or pDR196 vector [47], the non-Gateway version of pDR196-Ws as the control. The yeast cells were grown in liquid synthetic defined (SD) medium (1.7 g/L Yeast Nitrogen Base without nitrogen (Gibco, USA), 2 % glucose, 5 g/L NH_4_SO_4_, (Thermo Fisher Scientific, Waltham, MA) until they reached OD_600_ > 1, harvested by centrifuging, washed twice with sterile water, and diluted to OD600 = 1 in water. The cell suspensions were further diluted tenfold thrice to make the serial dilutions, then 5 μL of each dilution was spotted on the SD agar medium containing either amino acids or NH_4_SO_4_ as the sole nitrogen source. Yeast uptake assays were performed as previously described [25]. Competition assays were performed by incubating the yeast cells in 1 mM of ^3^[H] labeled amino acids (final specific activity of 252 kBq µmol^−1^) and 5 mM of competing, unlabeled amino acids.

### 4.5. Transient Expression of GFP Fusions in Nicotiana Benthamiana

PGGZ-ClAAP3-GFP and pGGZ-ClAAP6-GFP plasmids were each co-transfected with pSOUP vector into *Agrobacterium tumefaciens* GV3101 using a standard electroporation method. pGWB506-GFP-ClAAP fusions were transformed into *Agrobacterium tumefaciens* GV3101 using a standard electroporation method. Transient expression in *Nicotiana benthamiana* was performed as previously described [48]. Briefly, *Agrobacterium* carrying ClAAP3-GFP, ClAAP6-GFP, or mCherry-HDEL [28] were resuspended in infiltration buffer (10 mm MgCl_2_, 10 mm MES pH5.6, 150 μm acetosyringone, Thermo Fisher Scientific, Waltham, MA) at OD = 0.6, incubated for one hour at room temperature, then injected into the abaxial side of *Nicotiana benthamiana* using a 1-mL syringe.

### 4.6. Transient Expression of GFP Fusions in Protoplasts Isolated from Watermelon Fruits

Young watermelon fruits (~10 DAP) were sliced thinly using a razor blade, and the sections were incubated in the wall digesting solution (500 mM mannitol (Thermo Fisher Scientific, Waltham, MA), 10 mM CaCl_2_, 20 mM KCl, 20 mM MES-KOH (Thermo Fisher Scientific, Waltham, MA), 1% BSA, 1% Cellulase Onozuka (Yakult, Tokyo, Japan), 0.5% Marcerozyme 10 (Yakult, Tokyo, Japan), and 0.1% β-mercaptoethanol at pH 5.6 (TCI America, Portland, Oregon) for 2.5 h. The protoplasts were filtered through a 70-μm mesh and transformed with the GFP fusion constructs, as described in [49].

### 4.7. Confocal Microscopy

ClAAP–GFP fusions were imaged using an Olympus^®^ FV1000 confocal microscope Olympus, Waltham, MA, USA) equipped with a UPLSAPO 60×/1.2 water immersion with a confocal pinhole size corresponding to 1 Airy unit with an appropriate filter set for GFP (Excitation 488 nm, Emission 500–530 nm). Zoom and image size was adjusted to ensure Nyquist sampling (pixel size 100 nm).

### 4.8. Total RNA Extraction from Watermelon Tissues and qRT-PCR

Total RNA was extracted from watermelon fruit tissue in three biological replicates using RNeasy^®^ Plant Mini Kit (QIAGEN Sciences, Germantown, MD, USA) as per the manufacturer’s protocol. The first strand cDNAs were synthesized with 1 μg of total RNA in 20 μL of the reaction mixture using iScript RT Supermix (Bio-Rad Laboratories, Inc, Hercules, CA, USA) as per the manufacturer’s instructions. Gene expression analysis via reverse transcription-qPCR was performed using the BioRad CFX96 qPCR instrument and the SsoAdv Univer SYBR GRN Master Kit (Bio-Rad Laboratories, Inc, Hercules, CA, USA). The watermelon β-actin was used as an internal control, and the relative expression levels (Cq values) for each gene were normalized to β-actin transcription. The relative expression levels were calculated using the ΔΔCq (quantitative cycle) method provided with the Bio-Rad CFX software. The primers used for qRT-PCR analysis are listed in Table S1.

## 5. Conclusions

Our study shows that ClAAP3 and ClAAP6 Are broad-spectrum amino acid transporters in watermelon. ClAAP3 expression increases in mature fruits, corresponding to the stage at which Arg and Cit accumulation takes place. ClAAP3 has a high affinity to Arg, suggesting that it might play a role in the accumulation of Arg and other amino acids in maturing fruits. ClAAP6 expression is higher in younger fruits and preferred neutral over basic (e.g., Arg, Lys, and His) amino acids. Therefore, the two transporters are likely to play distinct roles in amino acid transport into developing watermelon fruits. Future studies using gene editing will be desirable to interrogate whether these transporters are involved in the dynamic accumulation of amino acids in watermelon or other cucurbitaceous crops.

## Figures and Tables

**Table 1:** Putative amino acid transporters from watermelon. LHT: lysine histidine transporter; ProT: proline transporter; GAT: GABA transporter; AAP: amino acid permeases; ATLa: amino acid transporter-like a; ATLb: amino acid transporter-like b; AUX: auxin transporter; LAX: like AUX; PUT; putrescine transporter; CAT: cationic amino acid transporter.

LHT (8)	ProT (1)	GAT (4)	AAP (8)	ATLa (5)	ATLb (8)	LAX/AUX (7)	PUT (5)	CAT (8)
Cla006527	Cla004309	Cla020511	Cla005590	Cla010799	Cla000539	Cla015837	Cla005022	Cla003079
Cla004045		Cla020512	Cla023090	Cla009967	Cla015743	Cla020298	Cla001768	Cla003075
Cla009988		Cla011382	Cla013912	Cla015343	Cla012146	Cla000681	Cla007330	Cla006711
Cla019489		Cla010921	Cla023187	Cla007263	Cla017006	Cla017975	Cla016085	Cla013686
Cla020372			Cla011628	Cla007265	Cla002877	Cla018110	Cla002221	Cla019526
Cla011987			Cla011631		Cla002878	Cla006581		Cla012403
Cla020709			Cla011627		Cla020546	Cla004339		Cla005396
Cla015783			Cla011629		Cla013755			Cla002657

## Supplementary Materials

Supplementary materials can be found at https://www.mdpi.com/1422-0067/20/23/5855/s1.
