# Supplementary material for: Broad-Spectrum Amino Acid Transporters ClAAP3 and ClAAP6 Expressed in Watermelon Fruits
Source: Int J Mol Sci. 2019 Nov 22;20(23):5855. doi: 10.3390/ijms20235855 (PMC6928636; doi:10.3390/ijms20235855)
Supplement: Supplementary file 1 [file ijms-20-05855-s001.pptx]

## Slide 1
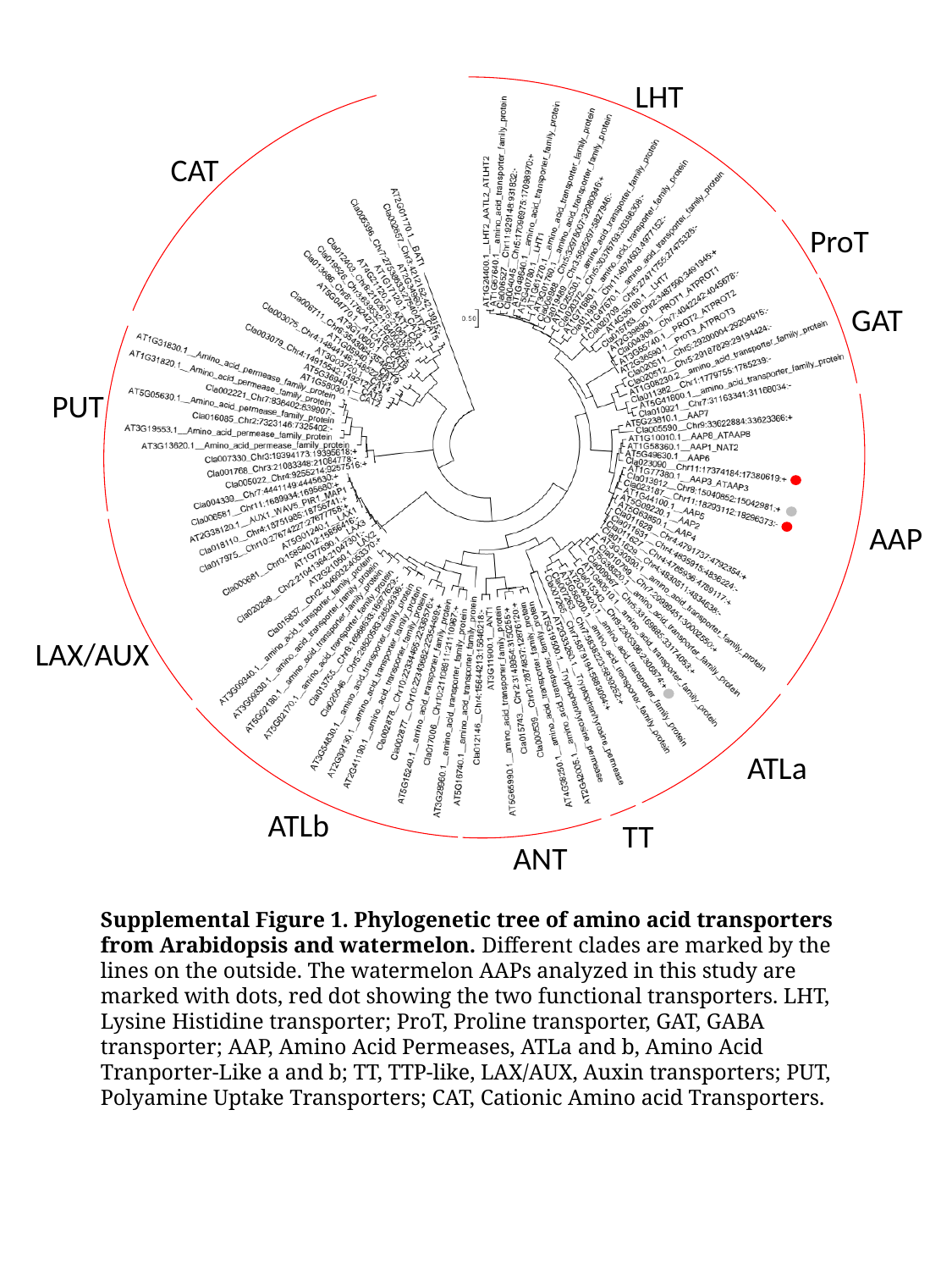

LHT
CAT
ProT
GAT
PUT
AAP
LAX/AUX
ATLa
ATLb
TT
ANT
Supplemental Figure 1. Phylogenetic tree of amino acid transporters from Arabidopsis and watermelon. Different clades are marked by the lines on the outside. The watermelon AAPs analyzed in this study are marked with dots, red dot showing the two functional transporters. LHT, Lysine Histidine transporter; ProT, Proline transporter, GAT, GABA transporter; AAP, Amino Acid Permeases, ATLa and b, Amino Acid Tranporter-Like a and b; TT, TTP-like, LAX/AUX, Auxin transporters; PUT, Polyamine Uptake Transporters; CAT, Cationic Amino acid Transporters.

## Slide 2
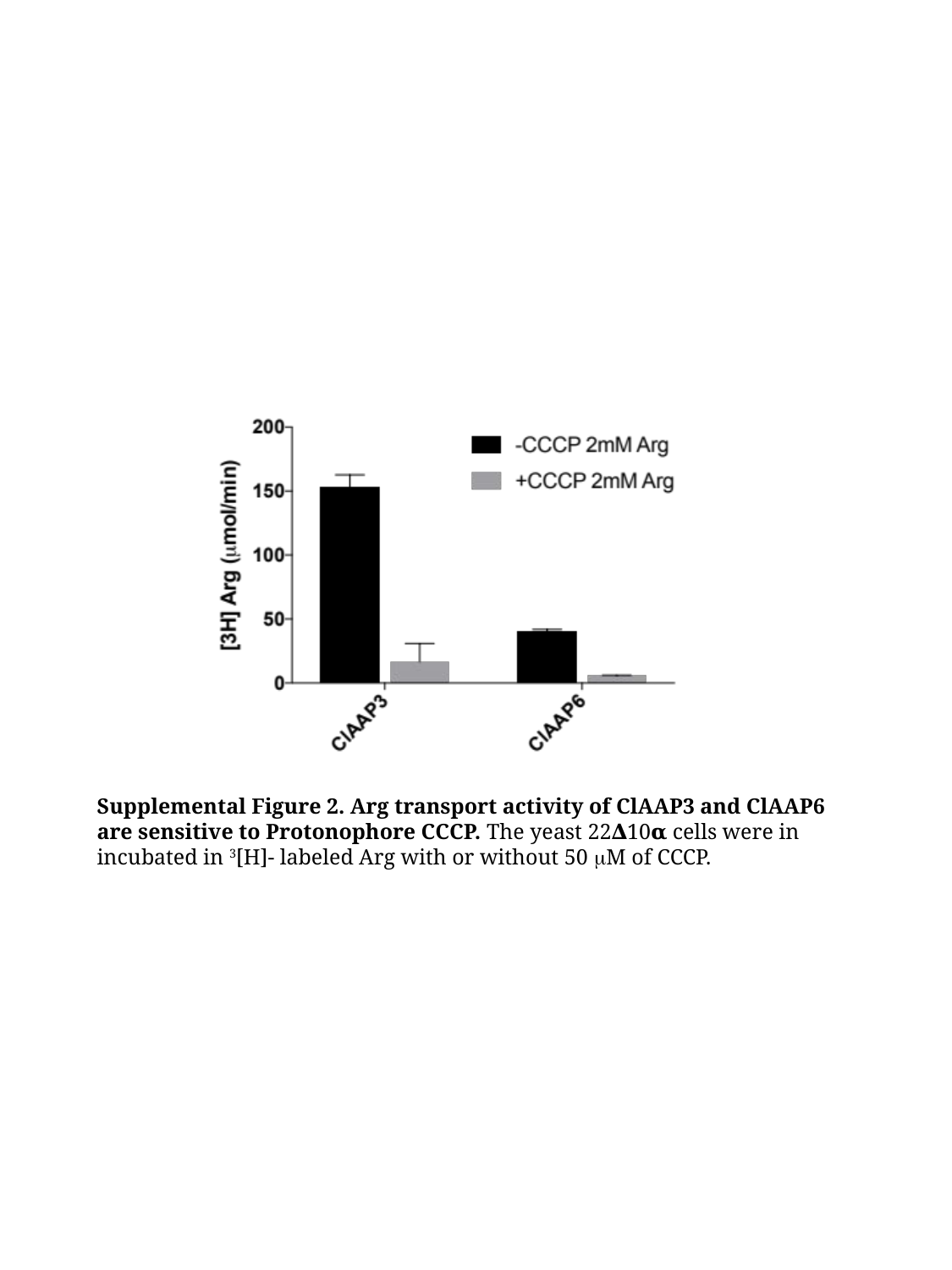

Supplemental Figure 2. Arg transport activity of ClAAP3 and ClAAP6 are sensitive to Protonophore CCCP. The yeast 22∆10𝛂 cells were in incubated in 3[H]- labeled Arg with or without 50 μM of CCCP.

## Slide 3
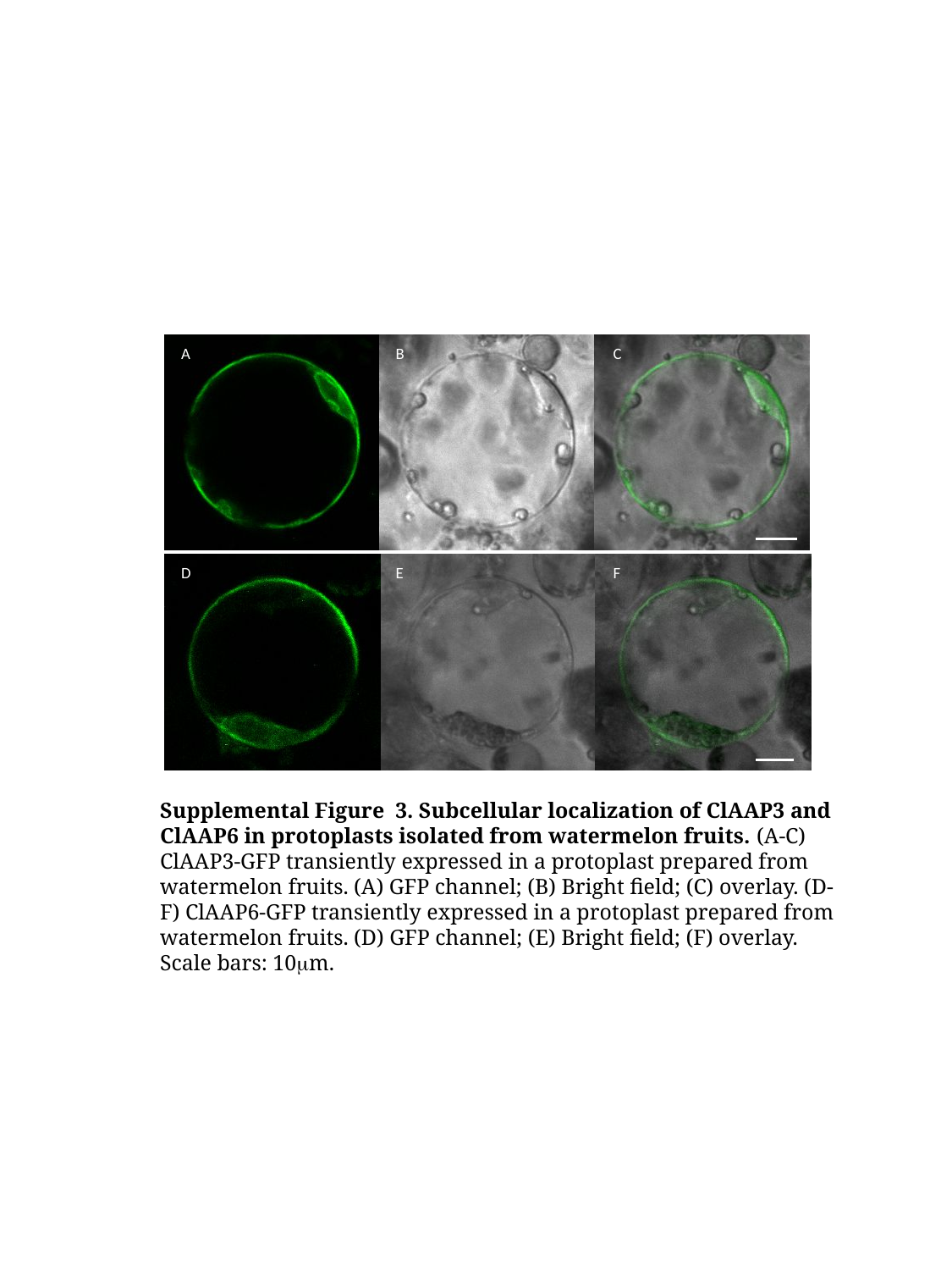

A
B
C
D
E
F
Supplemental Figure 3. Subcellular localization of ClAAP3 and ClAAP6 in protoplasts isolated from watermelon fruits. (A-C) ClAAP3-GFP transiently expressed in a protoplast prepared from watermelon fruits. (A) GFP channel; (B) Bright field; (C) overlay. (D-F) ClAAP6-GFP transiently expressed in a protoplast prepared from watermelon fruits. (D) GFP channel; (E) Bright field; (F) overlay. Scale bars: 10μm.

## Slide 4
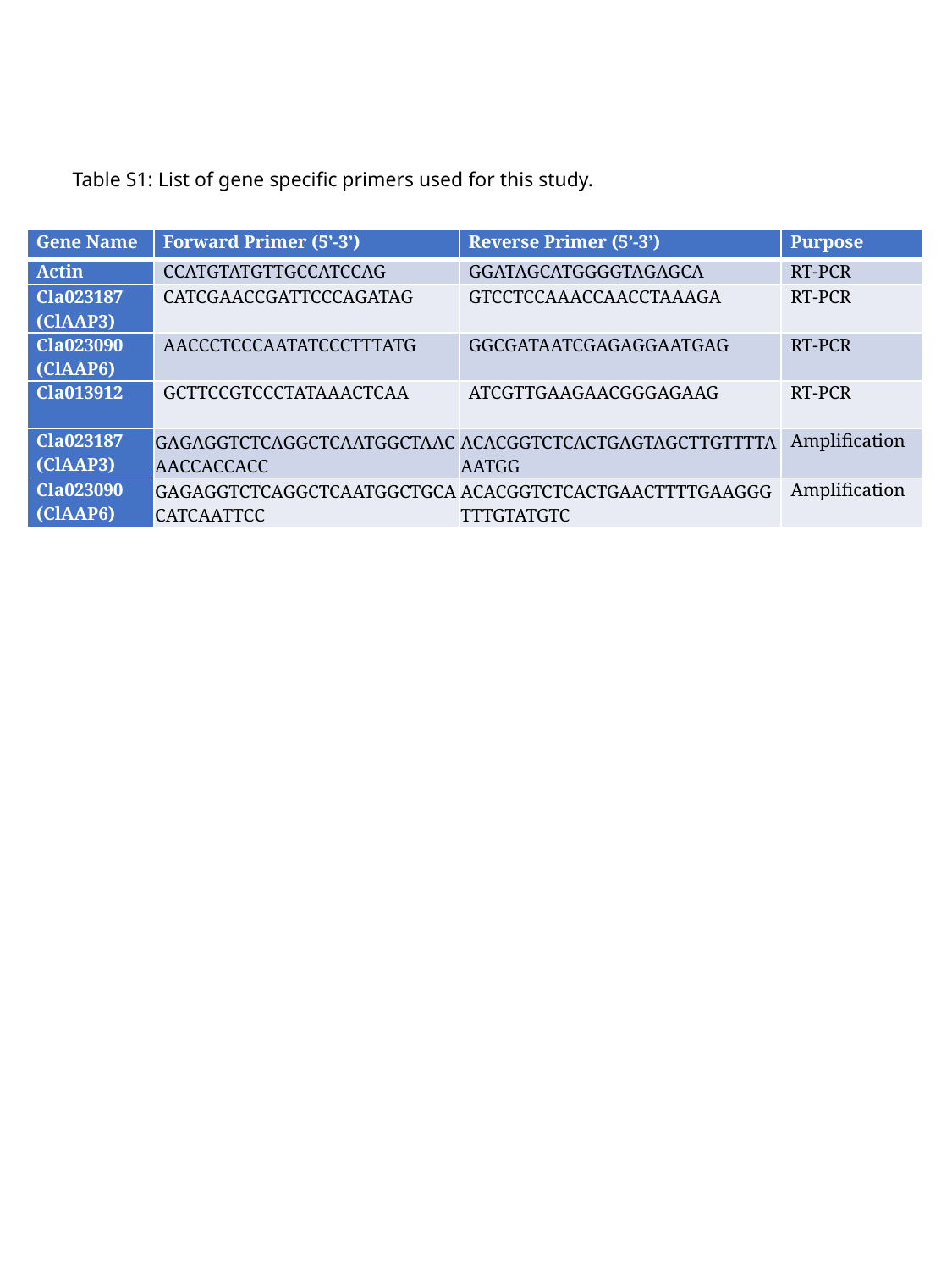

Table S1: List of gene speciﬁc primers used for this study.
| Gene Name | Forward Primer (5’-3’) | Reverse Primer (5’-3’) | Purpose |
| --- | --- | --- | --- |
| Actin | CCATGTATGTTGCCATCCAG | GGATAGCATGGGGTAGAGCA | RT-PCR |
| Cla023187 (ClAAP3) | CATCGAACCGATTCCCAGATAG | GTCCTCCAAACCAACCTAAAGA | RT-PCR |
| Cla023090 (ClAAP6) | AACCCTCCCAATATCCCTTTATG | GGCGATAATCGAGAGGAATGAG | RT-PCR |
| Cla013912 | GCTTCCGTCCCTATAAACTCAA | ATCGTTGAAGAACGGGAGAAG | RT-PCR |
| Cla023187 (ClAAP3) | GAGAGGTCTCAGGCTCAATGGCTAACAACCACCACC | ACACGGTCTCACTGAGTAGCTTGTTTTAAATGG | Amplification |
| Cla023090 (ClAAP6) | GAGAGGTCTCAGGCTCAATGGCTGCACATCAATTCC | ACACGGTCTCACTGAACTTTTGAAGGGTTTGTATGTC | Amplification |
